# Transformer-based approach for symptom recognition and multilingual linking

**DOI:** 10.1093/database/baae090

**Published:** 2024-09-11

**Authors:** Sylvia Vassileva, Georgi Grazhdanski, Ivan Koychev, Svetla Boytcheva

**Affiliations:** Faculty of Mathematics and Informatics, Sofia University St. Kliment Ohridski, Blvd “James Bourchier” 5, Sofia 1164, Bulgaria; Faculty of Mathematics and Informatics, Sofia University St. Kliment Ohridski, Blvd “James Bourchier” 5, Sofia 1164, Bulgaria; Faculty of Mathematics and Informatics, Sofia University St. Kliment Ohridski, Blvd “James Bourchier” 5, Sofia 1164, Bulgaria; Faculty of Mathematics and Informatics, Sofia University St. Kliment Ohridski, Blvd “James Bourchier” 5, Sofia 1164, Bulgaria; Ontotext, Ontotext, ul. “Nikola Gabrovski” 79, Sofia 1700, Bulgaria

## Abstract

This paper presents a transformer-based approach for symptom Named Entity Recognition (NER) in Spanish clinical texts and multilingual entity linking on the SympTEMIST dataset. For Spanish NER, we fine tune a RoBERTa-based token-level classifier with Bidirectional Long Short-Term Memory and conditional random field layers on an augmented train set, achieving an F1 score of 0.73. Entity linking is performed via a hybrid approach with dictionaries, generating candidates from a knowledge base containing Unified Medical Language System aliases using the cross-lingual SapBERT and reranking the top candidates using GPT-3.5. The entity linking approach shows consistent results for multiple languages of 0.73 accuracy on the SympTEMIST multilingual dataset and also achieves an accuracy of 0.6123 on the Spanish entity linking task surpassing the current top score for this subtask.

**Database URL:** https://github.com/svassileva/symptemist-multilingual-linking

## Introduction

Clinical named entity recognition (NER) and entity linking are the task of automatically detecting important terms in clinical text like symptoms, diseases, procedures, diagnoses, and others and identifying the correct concept from a standard medical ontology or classification represented by the text. Detecting and normalizing clinical terms is an important task in clinical Natural Language Processing used for extracting structured information from clinical texts and allowing its subsequent automatic processing for downstream tasks. Due to the complexity of clinical texts, extracting the terms and linking them to the correct concepts from medical ontologies is a very challenging task. The search space for entity linking is usually hundreds of thousands of concepts, and the majority of these concepts are in the long tail of the distribution, i.e. they have very few representatives in the train datasets and medical vocabularies. Transformer-based models have been successfully applied for entity recognition, especially in high-resource languages like English. However, due to the limited labeled resources in other languages, the task of clinical NER remains challenging for a lot of languages.

Different shared tasks are organized each year to advance the methods for clinical information extraction by providing reference datasets that different teams can use to train their methods. The BioCreative Challenge and Workshop presents researchers with different challenges and allows them to compete and present their work at the BioCreative Workshop (1).

SympTEMIST is a shared task, part of the BioCreative VIII Challenge, aimed at detecting and normalizing symptoms, signs, and findings in Spanish clinical texts (2). The organizers provided a Spanish clinical dataset for training and evaluation of the participants’ methods. It consists of three subtasks—NER (Subtask 1), entity linking (Subtask 2), and multilingual entity linking (Subtask 3) for several languages—English, Portuguese, French, Italian, and Dutch (https://temu.bsc.es/symptemist/). The shared task aims to identify all signs, symptoms, non-numerical descriptions of test results, findings from imaging procedures, as well as patient death events. The first subtask focuses on NER in Spanish clinical case reports of entities from the aforementioned categories. The second subtask consists of identifying the correct Systematized Nomenclature of Medicine-Clinical Terms (SNOMED CT) code associated with each mention from Subtask 1. SNOMED CT (https://www.nlm.nih.gov/healthit/snomedct/us_edition.html) is an internationally used medical ontology and collection of terms used for clinical documentation and reporting (3). The list of possible SNOMED CT codes is provided by the organizers, and it is possible for some mentions to be missing from the knowledge base (KB). The third subtask includes identifying the correct SNOMED CT code in five different languages for automatically transferred entities in 350 case reports. The target list of SNOMED CT codes is the same as in Subtask 2.

This paper describes our approach for symptom recognition in Spanish as well as multilingual entity linking in six languages. The contributions of this paper are as follows: we propose a system for Spanish clinical NER using a data augmentation approach which shows a 0.73 F1 score on symptom recognition. We propose a hybrid approach for improving entity linking in multiple languages using dictionaries, cross-lingual SapBERT generation, and GPT 3.5 reranking which shows a consistently high accuracy score of 0.73 on average across five languages, improving on the best score on the SympTEMIST dataset (4). The approach can be adapted to other languages as well. We have published our code in GitHub (https://github.com/svassileva/symptemist-multilingual-linking).

## Related work

Approaches based on transformer architectures are frequently utilized for NER tasks. Notably, the top-ranked system in the SympTEMIST challenge incorporates an ensemble of transformer models for Spanish clinical text and achieved an F1 score of 0.74 (strict) (5). Prior research on NER for Spanish text demonstrated that Spanish RoBERTa (6) enhanced with a conditional random field (CRF) head outperforms other NER models (7, 8). Moreover, the integration of a Bidirectional Long Short-Term Memory (BiLSTM) layer has been shown to improve model performance, achieving an F1 score of 0.79 in the context of the procedure challenge (7).

The widely adopted solution for entity linking in the context of clinical texts is the application of cosine similarity search alongside embeddings from the cross-lingual SapBERT model, as introduced by Liu et al. (9). Such an approach was utilized by the top three teams in the MedProcNER challenge (https://temu.bsc.es/medprocner/) (10, 11, 8), as well as by top-ranked teams in the SympTEMIST challenge, augmented with a reranking model based on Bidirectional Encoder Representations from Transformers (BERT), achieving an accuracy of 0.60.

The most prominent multilingual end-to-end solution for NER and entity linking is mReFinED (12). The proposed two-state solution is based on a semisupervised method for annotating entity mentions within Wikipedia articles, subsequently leveraging this annotated corpus to train a multifaceted BERT-oriented model performing NER, entity disambiguation, and entity linking.

Some multilingual general domain approaches based on sequence-to-sequence models successfully utilize entity linking, as exemplified by the mGENRE system (13). The latter one is designed to predict the normalized entity name for each mention in a specified language using an auto-regressive model (13) and demonstrated state-of-the-art results for the benchmark dataset Mewsli-9 (14).

In the biomedical and clinical domain, Zhu et al. propose a system that maps language-specific mentions to Unified Medical Language System (UMLS) using a controllable contrastive generation framework using a template-based UMLS concept summary to guide the decoder to generate the correct entity (15).

## Data

### SympTEMIST dataset

The SympTEMIST corpus (2) contains 1000 clinical case reports in Spanish with labeled symptom spans and their corresponding concept code from SNOMED CT. The corpus contains labeled train and test sets and a gazetteer of SNOMED CT codes and different aliases in Spanish. The train set has 750 documents, 11,899 sentences, and 343,243 tokens. The test set has 250 documents, 3,986 sentences, and 114,536 tokens. The train set contains 3,484 annotated entities with 1534 unique entity codes. Fifty-nine mentions have no SNOMED CT code assigned, and the rest have a single corresponding code. An additional set of composite mentions was released after the end of the challenge. There is one nested mention. The Spanish SympTEMIST gazetteer contains a total of 164,817 aliases for terms in multiple categories, including findings, disorders, morphologic abnormalities, and others.

**Figure 1.**
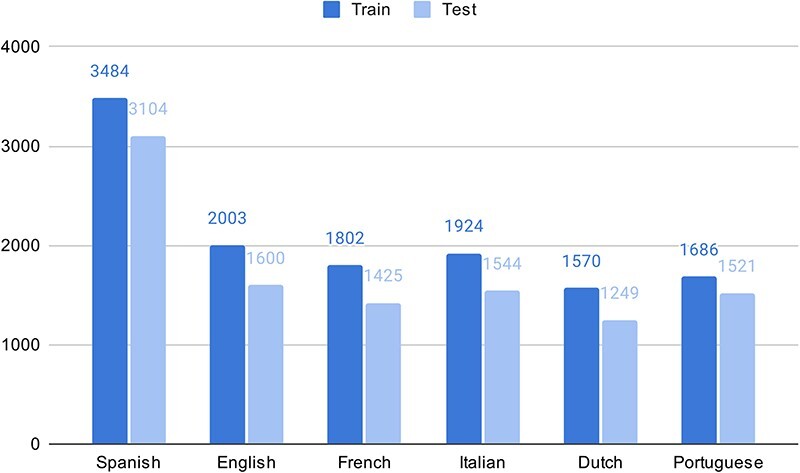
The number of entities in the train and test sets in different languages in the SympTEMIST dataset.

**Figure 2.**
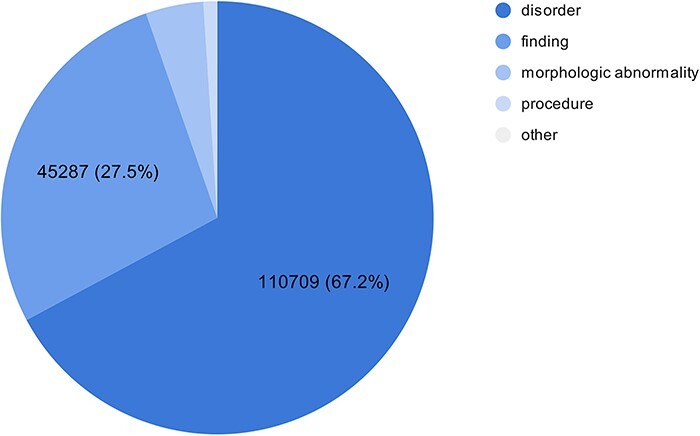
The number of concepts per type in the gazetteer in the SympTEMIST dataset.

As part of the experimental multilingual subtask, the organizers automatically translated 350 Spanish clinical case reports into five languages and then transferred the labeled entities from Spanish into the target language using lexical annotations transfer techniques (2). The goal is to predict the correct SNOMED CT code based on the identified entity mentioned in the text. Figure 1 shows the number of entities in the train and test sets in different languages in the SympTEMIST dataset. Due to differences in the automatic translation quality, the number of labeled entities is slightly different in the different languages (2). Figure 2 shows the distribution of SNOMED CT concept types in the SympTEMIST gazetteer. The majority of concepts are disorders, followed by findings and morphologic abnormalities.

### Language pretraining dataset

To evaluate the effect of further pretraining of the language model, we compiled an additional dataset consisting of Spanish UMLS synonyms of all terms included in the SympTEMIST gazetteer, for a total of 337,039 aliases.

### UMLS KB

**Figure 3.**
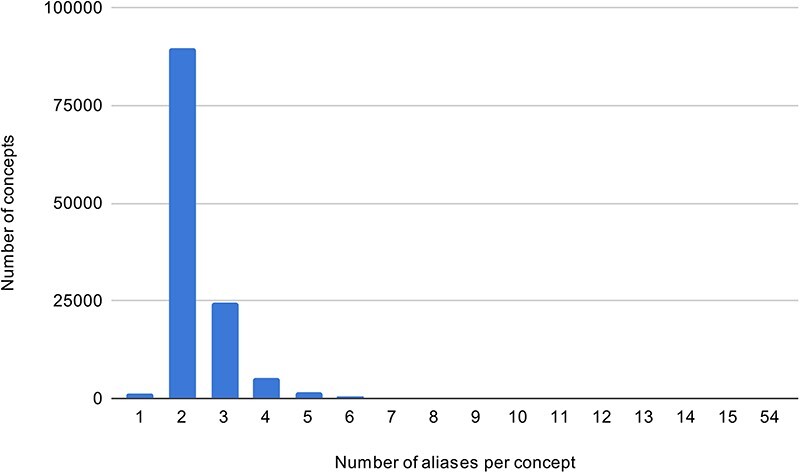
The concept alias frequency in the Spanish KB.

Using the UMLS Spanish SNOMED CT (3), a KB of all aliases of SympTEMIST gazetteer concepts was created. In addition, data from the gazetteer and the train set for Subtask 2 were added. The KB consists of 289,734 aliases of symptoms. Figure 3 shows the frequency distribution of concept aliases in the Spanish KB—the majority of concepts have up to two aliases.

**Figure 4.**
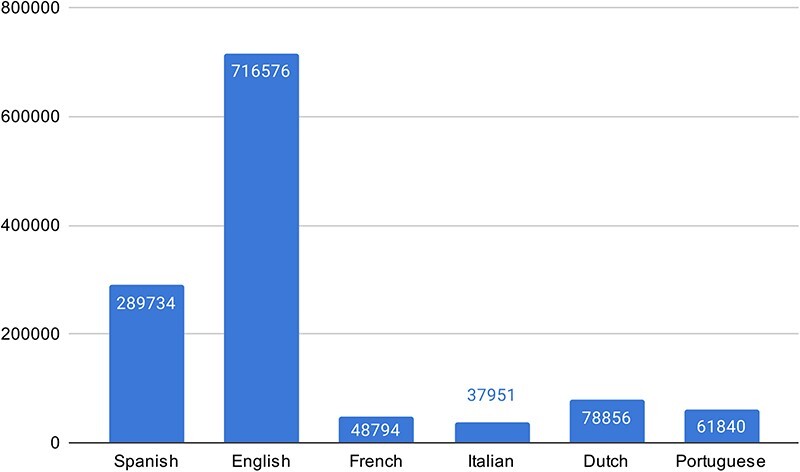
The number of aliases in the KB for the different languages.

For the multilingual subtask, we compiled all UMLS SNOMED CT aliases for the different languages and combined them with the train set entities. Figure 4 shows the number of aliases in the KB for each language.

## Methods

### Subtask 1—NER

**Figure 5.**
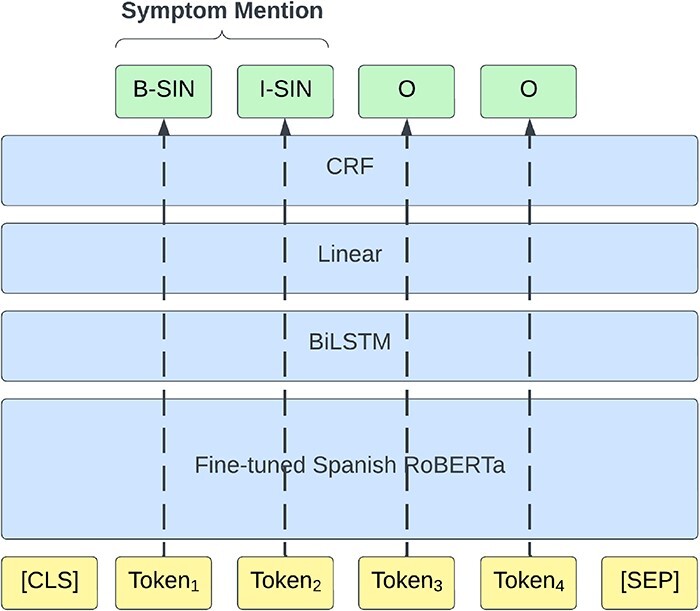
The architecture of the NER model.

The clinical report texts are first split into sentences using the SPACCC Sentence Splitter (https://github.com/PlanTL-GOB-ES/SPACCC_Sentence-Splitter) as about 33% of the case reports exceed the 512 input token limit of the employed models. Following the method we used in the original competition (16), we approach the NER subtask as a token classification task and label the train set using the IOB2 scheme (17). We train a transformer-based model with an additional two-layer BiLSTM, followed by a linear layer and a conditional random field (CRF) on top of the token classification task using the negative conditional log likelihood of a sequence of labels as a loss function. Figure 5 shows the NER pipeline architecture. We trained the model for 20 epochs using the following hyperparameters: learning rate, 5e-5 (recommended by the original BERT paper (18)); Adam beta1, 0.9; Adam beta2, 0.999 [Adam default configuration of huggingface library (https://huggingface.co/docs/transformers/en/main_classes/optimizer_schedules)]; weight decay, 0.1 (recommended by the original RoBERTa paper (19)); batch size, 8; and gradient accumulation steps, 2.

After splitting the training dataset into sentences, we augment it by randomly replacing some of the annotated mentions with a synonym from the Spanish UMLS. This results in 1672 additional example sentences for a total of 13,571.

#### Classification model selection

For the token classifier base model, we performed experiments with different Spanish BERT-based models—CLIN-X-ES (20) and PlanTL-GOB-ES/roberta-base-biomedical-clinical-es (6) (Spanish RoBERTa). We chose these models as they had performed well in previous challenges on NER in Spanish clinical texts showing competitive results in MedProcNER (21) and DisTEMIST (22).

CLIN-X-ES is based on XLM-RoBERTa Large, a cross-lingual language model, and is additionally trained on Spanish clinical corpus using the masked language modeling objective. The corpus combines the MeSpEN (23) dataset and Scielo archive (https://scielo.org/). We use this model as-is without further language pretraining.

Spanish RoBERTa (PlanTL-GOB-ES/roberta-base-biomedical-clinical-es) is a monolingual Spanish model, based on RoBERTa and trained on a large Spanish biomedical–clinical corpus of more than 1B tokens. Systems using this model have achieved very good results on previous Spanish biomedical–clinical tasks. We further pretrain the Spanish RoBERTa model on the language pretraining dataset to improve the language model’s training on symptoms.

#### Language model pretraining

We evaluate the effect of further pretraining of the base transformer model using the language pretraining dataset and the Spanish RoBERTa model with the masked language modeling objective for four epochs. Hyperparameter values are the same as those used for pretraining RoBERTa (base) in the original RoBERTa paper(19).

### Subtask 2—entity linking

The entity linking task aims to predict the correct SNOMED CT code, using the gold entities provided in the SympTEMIST dataset. The dataset originally provided by the organizers had all composite mentions, which correspond to more than one code, removed from the train and test sets, and therefore our system targets outputting a single code. Some entity mentions have no corresponding code in SNOMED CT and are marked as NO_CODE (59 mentions in the train set).

**Figure 6.**
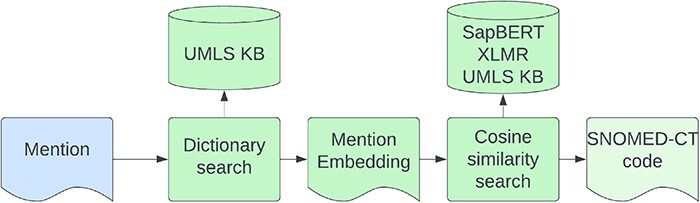
The architecture of the Enity Linking model.

We use the model developed for the competition (16) which did not have any composite mentions in its training data and therefore does not address the composite code problem. Entity linking is performed in two steps—first, we try to match the mention to an alias in the KB by using an exact match string search of the lowercase symptom name. Second, for entities that did not match a KB alias, we performed a cosine similarity search on the cross-lingual SapBERT (9) embeddings, retrieving the closest alias from the KB. The architecture of the entity linking model is shown in Figure 6. We gather symptom synonyms from different data sources including the SympTEMIST gazetteer, train data, and UMLS symptoms (3). We also augment the rare symptom concepts with less than five aliases by adding/removing random characters to generate five new aliases for each concept.

### Subtask 3—multilingual entity linking

**Figure 7.**
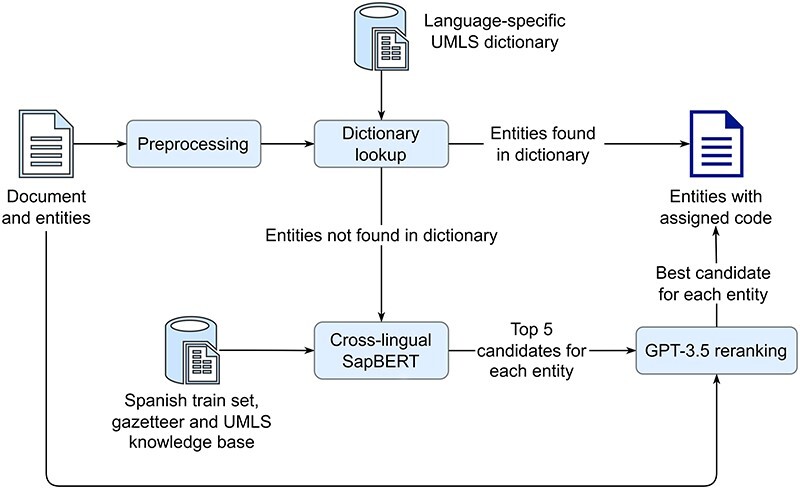
A pipeline for assigning SNOMED CT codes to automatically translated symptom mentions (Subtask 3), consisting of four main steps: first, symptom mentions are preprocessed and a dictionary lookup is performed, second if a mention is found in the dictionary, it is assigned the corresponding code, third, any mentions not found in the dictionary are processed by the cross-lingual SapBERT to find the five most similar candidates in a KB, and finally, for each mention, GPT-3.5 is prompted to determine the best candidate from the five, using the mention and the original Spanish document from which the mention is derived.

For the multilingual entity linking task, the organizers provided automatically translated symptom mentions in English, French, Italian, Dutch, and Portuguese with the corresponding SNOMED CT codes, that are derived from the original gold Spanish annotations, used in the second task. The pipeline used to assign a SNOMED CT code to the automatically translated mentions is shown in Figure 7. First, each mention is transformed to lowercase. Then, a lowercase exact match string search is performed against a language-specific dictionary of all UMLS SNOMED CT aliases, combined with entities from the corresponding train set. If a mention is found in the dictionary, it is assigned the corresponding code. Mentions not found in the dictionary are further processed by a cross-lingual SapBERT to find the five most similar entities (in terms of cosine similarity) from the UMLS KB. Finally, each mention, its five candidate entities, and the text of the Spanish patient case report from which the mention is derived are used to construct a one-shot prompt for GPT-3.5, which selects the best candidate. In case GPT-3.5 returns an entity that is not included in the five candidates provided, NO_CODE is assigned, which addresses a limitation of the basic cross-lingual SapBERT approach.

## Experiments and results

### Train and validation datasets

We compile the train and validation sets by first splitting the original texts into sentences and then creating three bins of sentences based on the longest mention contained in the sentence—short (less than 38 symbols), medium (between 38 and 90 symbols), and long (over 90 symbols). Finally, 80% of the sentences in each bin are used as train examples, and the rest—for validation. Bin margins are determined based on the mention length distribution in the original train set. The validation set is used for model comparison in different experiments, while the full train set is used to train the final models for test set evaluation.

Micro-averaged precision, recall, and F1-score are used for the NER subtask. Accuracy is used for entity linking.

### Subtask 1—NER

We split the train set into sentences and use 80% for training and 20% for validation. NER performance is measured using micro-averaged precision, recall, and F1-score.

Table 1 presents the results of the models and fine-tuning combinations that were submitted in the original challenge. Models based on the Spanish RoBERTa (PlanTL-GOB-ES/roberta-base-biomedical-clinical-es) perform best, likely because it is specialized for the Spanish biomedical–clinical domain. The addition of a two-layer BiLSTM increases both recall and precision, perhaps due to its ability to consider long-term dependencies(7). Table 2 shows the strict vs overlapping precision, recall, and F1 scores on the test set. The overlapping scores are significantly higher than the strict by 13%–15%, which could be explained by differences in the tokenization rules used by the annotators and our models. The augmented Spanish RoBERTa + BiLSTM + CRF model shows an overlapping F1 score of 0.8683 ranked second in the competition.

**Table 1.** Subtask 1 results on the validation and test sets of the models and fine-tuning combinations that were submitted in the original challenge (best scores are shown in bold) (Subtask 1—NER section)

Model	Val *P*	Val *R*	Val F1	Test *P*	Test *R*	Test F1
Spanish RoBERTa + CRF	0.730	**0.746**	0.738	-	-	-
Augmented Spanish RoBERTa + CRF	**0.773**	0.729	**0.750**	0.732	0.718	0.725
Augmented Spanish RoBERTa + BiLSTM + CRF	0.744	**0.732**	0.738	**0.739**	**0.725**	**0.732**
Pre-trained augmented Spanish RoBERTa + CRF	0.749	0.721	0.735	0.715	0.720	0.718
CLIN-X-ES + CRF	0.757	0.717	0.737	0.718	0.703	0.710
Augmented CLIN-X-ES + CRF	0.722	0.704	0.713	0.724	0.699	0.712

Models based on the Spanish RoBERTa perform best. The addition of a two-layer BiLSTM increases both recall and precision. The best score on the test set is achieved by the Augmented Spanish RoBERTa with BiLSTM and CRF layers—0.732 F1.

**Table 2.** Subtask 1 results on the test sets of the models submitted in the challenge—strict and overlapping precision, recall, and F1 scores (best scores are shown in bold) (Subtask 1—NER section).

Model	Test *P*	Test *R*	Test F1	Test overlap *P*	Test overlap *R*	Test overlap F1
Augmented Spanish RoBERTa + CRF	0.7324	0.7178	0.725	0.8702	0.8528	0.8614
Augmented Spanish RoBERTa + BiLSTM + CRF	**0.7393**	**0.7255**	**0.7324**	**0.8766**	0.8602	**0.8683**
Pretrained augmented Spanish RoBERTa + CRF	0.7149	0.7207	0.7178	0.8603	**0.8673**	0.8638
CLIN-X-ES + CRF	0.7245	0.6991	0.7116	0.8748	0.8441	0.8592
augmented CLIN-X-ES + CRF	0.7177	0.7026	0.7101	0.8651	0.847	0.8559

The overlapping scores are significantly higher than the strict by 13%–15% which could be explained by differences in the tokenization rules used by the annotators and our models. The Augmented Spanish RoBERTa + BiLSTM + CRF model shows an overlapping F1 score of 0.8683 which ranked second in the competition.

#### Effect of data augmentation

We compare the models trained on the original and the augmented dataset which contains 15% more examples generated by replacing symptom mentions with their UMLS synonyms. The model results on the augmented dataset are shown in Table 1. Using the augmented train set significantly improves the precision and F1 of the Spanish RoBERTa model after fine-tuning. A small performance drop is observed in the augmented CLIN-X-ES model compared to its nonaugmented version on the validation set. However, on the test set, the two models show very close F1 scores, with the augmented CLIN-X-ES having a small precision lead.

#### Effect of language pretraining

Further pretraining of the Spanish RoBERTa degrades model performance when fine tuned on the augmented training set. Using only UMLS term synonyms out of context appears to be insufficient for conditioning the model for the NER task.

### Subtask 2—entity linking

For the Spanish entity linking task, we experiment with various KBs by combining the different resources—gazetteer, train set, and the UMLS synonym dataset. In addition, we augment the knowledge by adding/removing random characters in the aliases of rare concepts that have fewer than five records in the KB. For each rare concept, five new records are added to the KB. For validation purposes, we use the full train set and exclude examples that exactly match aliases in the KB. Accuracy is used to measure the entity linking performance.

We noticed that the straight-forward cosine similarity search using cross-lingual SapBERT embeddings struggles with longer mentions, so we performed an experiment tackling this challenge using a sliding window approach. For each alias in the KB, we determine the final similarity score as a linear combination of cosine similarities of three parts—the full mention, the first 75% of tokens in the mention, and the last 75% of tokens in the mention. Again, we select the KB alias with the highest combined cosine similarity score. Using grid search over the train set, we identify the optimal coefficients for the three parts in the linear combination to be 0.75, 0.17, and 0.08, respectively. This approach achieves 2% higher accuracy than the basic cross-lingual SapBERT model on the same KB, suggesting that the information needed to find the correct code is more focused in the first part of the mention.

The results for the entity linking subtask submitted in the challenge are presented in Table 3. The best model we submitted for the challenge achieved an accuracy of 0.589 on the test set and has the richest KB with additional data from UMLS. The majority of the models show close accuracy results in the range of 0.56–0.58. We also measured the effect of performing a dictionary match before the cosine similarity search. Excluding the dictionary matching resulted in lower scores of 1.4% on the validation set and 0.3% on the test set. There was a bug in the code for generating the predictions of this model, and it scored 0.01 in the official evaluation. After the challenge ended, we fixed the bug and reran the test, resulting in an accuracy of 0.586 on the test set.

**Table 3.** Subtask 2 results on the validation and test sets of the models submitted in the challenge show that the best model achieved an accuracy of 0.589 on the test set, using a UMLS-enriched KB (best scores are shown in bold) (Subtask 2—entity linking section).

Model	KB	Val	Test
		Acc	Acc
Cross-lingual SapBERT	Gazetteer + Train	0.514	0.588
Cross-lingual SapBERT	Gazetteer + Train + Aug.	0.533	0.565
Cross-lingual SapBERT	Gazetteer + Train + UMLS	0.524	**0.589**
Cross-lingual SapBERT + sliding window	Gazetteer + Train + Aug.	**0.536**	0.587
Cross-lingual SapBERT without dictionary match	Gazetteer + Train + UMLS	0.510	0.017/0.586*

The majority of the models show close accuracy results in the range of 0.56–0.58. Excluding the dictionary match from the pipeline resulted in a lower score on both validation and test sets. $^{\ast}$ The first result is the original submission which had a bug in the code, the second—after the bugfix post-competition.

### Subtask 3—multilingual entity linking

As a baseline for the multilingual entity linking task, we perform a lowercase exact match string search within the KB for each language. Table 4 includes the baseline results and the best results across languages, including Spanish (part of Subtask 2). All experiments for this subtask were performed after the challenge ended.

**Table 4.** Subtask 3 results on the test set for different languages after the challenge ended (best scores are shown in bold) (Subtask 3—multilingual entity linking section).

Model	Preprocessing	Language	Test set accuracy
Dictionary	Lowercase mentions	French	0.527
Dictionary + Cross-lingual SapBERT	-	French	0.708
Dictionary + Cross-lingual SapBERT + GPT 3.5 Reranking	-	French	0.7143
Dictionary + Cross-lingual SapBERT	GPT-3.5 Spanish translation	French	0.708
Dictionary + Cross-lingual SapBERT + GPT 3.5 Reranking	GPT-3.5 Spanish translation	French	**0.7284**
Dictionary	Lowercase mentions	English	0.596
Dictionary + Cross-lingual SapBERT	-	English	0.7362
Dictionary + Cross-lingual SapBERT + GPT 3.5 Reranking	-	English	0.7437
Dictionary + Cross-lingual SapBERT	GPT-3.5 Spanish translation	English	0.7462
Dictionary + Cross-lingual SapBERT + GPT 3.5 Reranking	GPT-3.5 Spanish translation	English	**0.7525**
Dictionary	Lowercase mentions	Dutch	0.549
Dictionary + Cross-lingual SapBERT	-	Dutch	0.7261
Dictionary + Cross-lingual SapBERT + GPT 3.5 Reranking	-	Dutch	0.7309
Dictionary + Cross-lingual SapBERT	GPT-3.5 Spanish translation	Dutch	**0.7365**
Dictionary + Cross-lingual SapBERT + GPT 3.5 Reranking	GPT-3.5 Spanish translation	Dutch	0.7301
Dictionary	Lowercase mentions	Italian	0.523
Dictionary + Cross-lingual SapBERT	-	Italian	0.6943
Dictionary + Cross-lingual SapBERT + GPT 3.5 Reranking	-	Italian	0.7156
Dictionary + Cross-lingual SapBERT	GPT-3.5 Spanish translation	Italian	0.713
Dictionary + Cross-lingual SapBERT + GPT 3.5 Reranking	GPT-3.5 Spanish translation	Italian	**0.726**
Dictionary	Lowercase mentions	Portuguese	0.503
Dictionary + Cross-lingual SapBERT	-	Portuguese	0.7047
Dictionary + Cross-lingual SapBERT + GPT 3.5 Reranking	-	Portuguese	**0.7271**
Dictionary + Cross-lingual SapBERT	GPT-3.5 Spanish translation	Portuguese	0.7094
Dictionary + Cross-lingual SapBERT + GPT 3.5 Reranking	GPT-3.5 Spanish translation	Portuguese	0.7251
Dictionary + Cross-lingual SapBERT + GPT 3.5 Reranking	-	Spanish	**0.6123**

The baseline dictionary approach shows the lowest score of 0.5396 on average across all languages. Enhancing the dictionary with a cosine similarity search using Cross-lingual SapBERT embeddings significantly improves the accuracy 14%–20% depending on the language. Using GPT-3.5 as a reranker of the final results makes additional improvements of 1%–2%, and finally preprocessing the original text and translating it into Spanish shows the best results for French, English, Italian, and Spanish.

For all languages, we use a cross-lingual SapBERT with the Spanish UMLS KB to assign a SNOMED CT code to symptom mentions that are not present in the dictionary. The model produces an embedding vector for the query symptom mention, which is then used to find the most similar (in terms of cosine similarity) entities from the KB. The Spanish UMLS knowledge was chosen, as it achieved our highest result in the second subtask while offering a good balance between the code variety and the number of aliases. For French, using the augmented KB instead leads to a lower accuracy score (0.682 vs 0.708 for the UMLS KB). Unsurprisingly, naively translating the KB into the target language (e.g. French) also causes performance degradation.

**Figure 8.**
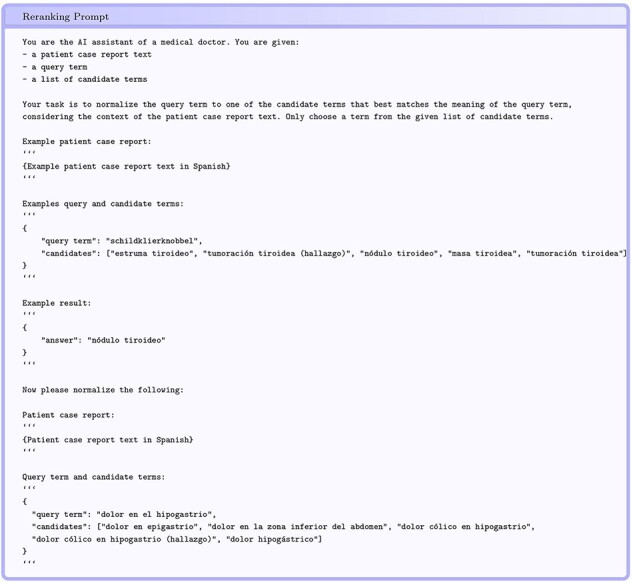
Example prompt for reranking candidate entities.

Figure 8 shows an example of the instructions we provide to GPT-3.5-turbo (gpt-3.5-turbo-0125) to rerank the candidates generated by the cross-lingual SapBERT. The prompt is constructed from a symptom mention, candidates from SapBERT, and the text of the patient report from which the symptom mention is derived. The example in the prompt varies between languages and is from the corresponding train set. The number of candidate mentions in the prompt has a significant impact on the reranking performance. Having fewer candidates is better (0.728 accuracy with five candidates for French), even though the right candidate could be found more often in the top 10 (0.712 accuracy with 10 candidates for French). Reranking performance is significantly affected by the presence of the correct entity among the top five candidate entities found by SapBERT. For English, the correct entity is in the top five candidates for about 0.52 of the entities that are processed by SapBERT and 0.76 of those are reranked correctly. Last but not least, the Spanish entity linking (Subtask 2) also benefits from the reranking step, achieving a new best accuracy score of 0.612 (achieved after the challenge deadline).

Translating the symptom mention from the original language into Spanish brings a stable performance improvement across languages. Since the KB constitutes UMLS terms in Spanish, it could explain why translating the search term in Spanish improves the system’s performance. Figure 10 shows the prompt used to translate a symptom mention into Spanish via GPT-3.5-turbo. Table 4 shows the impact of symptom mention translation and candidate reranking.

**Figure 9.**
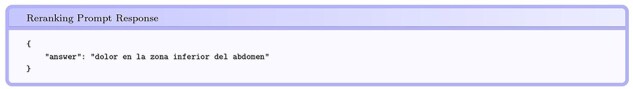
Example reranking result.

**Figure 10.**
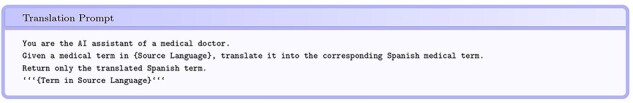
The prompt used for translating a symptom mention into Spanish.

## Error analysis

For the multilingual entity linking task, across all languages, about 2% of the test set symptom mentions match a term from the corresponding dictionary, but their code in the test set differs from the one in the dictionary. For example, in the Portuguese dictionary, the term *macrocitose* has a code of 397073000 (finding), while in the test set, it is assigned 72826005 (morphologic abnormality), both being valid candidate codes, depending on the context.

## Conclusion

We explore transformer-based approaches to solving the SympTEMIST NER and linking tasks. For NER, systems based on a specialized monolingual model achieve the best results. The addition of a BiLSTM layer after the last transformer layer, and train data augmentation, improves performance on the test set. Further pretraining on a UMLS synonyms dataset did not prove beneficial. For the entity linking task, we employ a hybrid approach, including UMLS dictionary matching, generating candidates using a cross-lingual SapBERT, and reranking the top five candidates using GPT-3.5. The choice of a KB has the highest impact on system performance—our highest accuracy model combines the SympTEMIST gazetteer, UMLS synonyms, and train set annotations. We performed experiments for entity linking with Spanish and five other European languages which showed a stable accuracy of 0.73 on average. The approach also improved the entity linking for Spanish and achieved an accuracy of 0.6123 on the test set, thus improving the current top score from the SympTEMIST challenge of 0.607.

All the challenge participants in the NER subtask use BERT-based models in some fashion, predominantly for token classification, and the best results are shown by ensemble models (5, 2). We could improve our system by training multiple BERT-based models in an ensemble. For entity linking, a lot of solutions use cross-lingual SapBERT but combine it in different ways. The best score on the entity linking challenge combines TF-IDF and SapBERT for candidate generation and a trained BERT-based cross-encoder for reranking (24). Our proposed method improves on their score but requires more compute resources and uses a proprietary model (GPT-3.5). As future work, we could explore fine-tuning an open-source model with less parameters like Llama3-8b (25) to rerank the suggested candidates which will allow the usage of the model in a lower-resource environment.

## Limitations

The entity linking experiments covered six different European languages which are covered by UMLS. Applying the same approach to languages from different language families or languages that are not represented in UMLS will probably result in lower performance. Since the dataset was automatically translated from Spanish and the annotations were automatically applied, the mentions that were successfully transferred may represent concepts that are easier to link since they are lexically similar. Therefore, the approach may not perform so well on a manually labeled dataset that includes “harder” mentions to link.
